# Astrocyte: A Foe or a Friend in Intellectual Disability-Related Diseases

**DOI:** 10.3389/fnsyn.2022.877928

**Published:** 2022-06-23

**Authors:** Busong Wang, Lu Zou, Min Li, Liang Zhou

**Affiliations:** Department of Pharmacology, College of Pharmaceutical Sciences, Soochow University, Suzhou, China

**Keywords:** astrocyte, intellectual disability, drug therapy, neurodevelopment, central nervous system

## Abstract

Intellectual disabilities are a type of neurodevelopmental disease caused by neurological dysfunction. Their incidence is largely associated with neural development. Astrocytes are the most widely distributed cells in the mammalian brain. Previous studies have reported that astrocytes only supported and separated the neurons in the brain. However, recent studies have found that they also play an important role in neural development. Understanding the astrocyte mechanism in intellectual development disorder-related diseases will help provide new therapeutic targets for the treatment of intellectual disability-related diseases. This mini-review introduced the association between astrocyte and intellectual disabilities. Furthermore, recent advances in genetic and environmental factors causing intellectual disability and different pharmaceutical effects of intellectual disability-related drugs on astrocytes have been summarised. Finally, we discussed future perspectives of astrocyte-based therapy for intellectual disability.

## Introduction

Astrocytes are widely distributed in the brain and are characterised as the largest glial cells in size, and their number in the brain is greater than the neurons (Sofroniew and Vinters, 2010). They are known as astrocytes because they resemble the stars. Astrocytes evolved from the original neural progenitor cells and then differentiated into two types of cells based on their different distributions in the brain: fibrous and protoplasmic astrocytes (Miller and Raff, 1984). Fibrous astrocytes are slender with few branches mainly distributed in the white matter of the brain marrow. Furthermore, fibrous astrocytes contain many glial filaments comprising glial fibrillary acidic protein (GFAP). However, protoplasmic astrocytes have more cytoplasmic branches and fewer glial filaments, which are mainly distributed in the grey matter (McGann et al., 2012). Based on single-cell sequencing, a recent study suggested that astrocyte might contain multiple subtypes with transcriptomic differences (Batiuk et al., 2020). These subtypes were brain region-specific with distinct morphological and physiological functions (Batiuk et al., 2020).

Astrocytes have molecular marker specificity. The GFAP is a specific molecular marker protein of astrocytes (Sticozzi et al., 2021). GFAP expression is closely associated with the astrocyte activation; therefore, the GFAP can be used as a reliable and sensitive marker of reactive astrocytes (Liddelow and Barres, 2017). Furthermore, S100β is an acidic calcium-binding protein synthesised and released by astrocytes that mainly affect the growth, proliferation and differentiation of glial cells (Donato et al., 2013). Similar to the GFAP, S100β is also considered a molecular marker of astrocytes (Cox et al., 2018). Upon astrocyte activation, the S100β expression also increases (Shobha et al., 2010). Vimentin (Vim) is also a member of the intermediate filament family (Terriac et al., 2017)and rarely expressed in astrocytes under normal conditions. However, in damaged CNS, the Vim expression will increase in reactive astrocytes, making Vima molecular marker of astrocytes (Ivaska et al., 2007). These molecular markers provide an important basis for investigating the roles and changes of astrocytes in the pathological state of the CNS.

Intellectual disability (ID) is a neurodevelopmental disease characterised by intellectual function and adaptability defects usually occurring during the developmental stages of neurons (Purugganan, 2018). It is the most common concomitant symptom of many diseases, such as Rett syndrome (RS), Down’s syndrome (DS), and fragile X syndrome (FXS) (Purugganan, 2018; Romano, 2022). The overall intelligence quotient of patients with ID is < 70 (Shea, 2012). Neuron growth retardation is the main cause of ID. In previous studies, neuronal morphology and functional changes were believed to be major factors inducing ID (McGann et al., 2012). In recent years, with the continuous research on astrocytes, astrocytes are found to play an important role in affecting the function of neural development (Cresto et al., 2019). Gene sequencing results in patients with ID show that a large number of ID-related genes are specifically expressed in astrocytes, not in the neurons (Lelieveld et al., 2016), suggesting that astrocytes, like neurons, are also important ID regulators.

Previous studies have suggested that astrocytes mainly develop around the neurons, and their processes can extend and fill the cell body and neurite to support and separate the neurons. In some astrocytes, the end process becomes the end feet, making it easier to adhere to the capillary wall or brain and spinal cord surfaces to form glial-limiting membranes (Hanani and Verkhratsky, 2021). With the continuous investigation of astrocytes, they have been found to regulate the central nervous system (CNS). Astrocytes can establish two-way communication with the neurons, participate in synaptic composition (Kofuji and Araque, 2021), respond to neurotransmitters released by synapses (Durkee and Araque, 2019), and become reactive upon chemical or physical insult. They could also be activated by the microglia and regulate neuronal and oligodendrocyte deaths (Liddelow et al., 2017). These reactive astrocytes are also involved in synaptic formation and clearance and synaptic plasticity regulation (Van Horn and Ruthazer, 2019). Furthermore, astrocyte ion channels are also involved in the neurotransmitter and ion metabolisms, such as glutamate and adenosine triphosphate (ATP) (Sofroniew and Vinters, 2010), regulating the neuronal excitability and maintaining the blood–brain barrier (Halassa et al., 2007). Taken together, these studies indicated that reactive astrocytes play a complicated role in the pathogenesis of ID. Therefore, this mini-review aimed to investigate the morphology and functional changes of reactive astrocytes in ID-related diseases and discuss the possible effects of drugs on the astrocytes.

## Astrocytes in Intellectual Disability

The factors causing ID can be divided into genetic and environmental (Table 1). Regarding the genetic factors, various genetic syndromes can result in intellectual impairment, such as DS, caused by chromosomal variation (another chromosome 21) (Chen et al., 2014), Williams syndrome caused by deletion of the region near the long-arm chromosome 7 (7q11.23) (Neuman and Henske, 2011), FXS caused by FMR1 gene defect and its protein product deletion (Pacey et al., 2015), RS caused by the MECP2 gene mutation on the X chromosome (Andoh-Noda et al., 2015), and tuberous sclerosis (TS) caused by TSC1 and TSC2 gene mutations (Neuman and Henske, 2011). As for the environmental factors, malnutrition, brain trauma, maternal perinatal infection, and early childhood CNS infection may cause an intellectual impairment (Purugganan, 2018). Several genetic diseases, including DS and FXS, are considered to be caused by neuronal changes (Fernández-Blanco and Dierssen, 2020). With an in-depth investigation of astrocytes, the pathological roles of astrocytes in neurodevelopmental disorders have been continuously explored, suggesting that changes in astrocytes may play an important role in the pathogenesis of ID.

**TABLE 1:** Factors associated with intellectual disability (Purugganan, 2018).

Factors leading to intellectual disability					
	**Genetic and environmental factors**		**Pathogenic mechanism of abnormal genes**	**Developmental process**	**Common symptoms**
Genetic factors	Chromosome abnormality	Down syndrome	Trisomy 21 (95%) Translocation (4%) Mosaicism (1%)	Mild to moderate ID (verbal-low) Hypotonia Early Alzheimer’s disease	Down facies Congenital heart disease Hypothyroidism Gastrointestinal abnormalities
	Contiguous gene deletion	Williams syndrome	7q11 deletion	Mild to moderate ID (nonverbal-low)	Elfin-like face Cardiovascular symptoms and (e.g., supravalvular aortic stenosis) Hypertension
	Single-gene deletion	Fragile X syndrome	CGG trinucleotide repeat(> 200)	Moderate ID Learning disorder Autism spectrum disorder	Elongated face Macrocephaly Prominent ear Enlarged testes
		Rett syndrome	MeCP2 deletion	ID Language regression	Head growth retardation Gait abnormalities
Environmental factor	Alcohol and other teratogenic factors, pregnancy infection, early childhood central nervous system infection, brain trauma, severe malnutrition and chronic iron deficiency				

From an in-depth investigation of the morphology and function of glial cells, the significance of astrocyte morphological and functional abnormalities to the occurrence of intellectual impairment gradually emerged (Cresto et al., 2019). For example, early experiments showed that RS was caused by the MeCP2deletion in neurons (Giacometti et al., 2007; Guy et al., 2007); however, in a recent study, Ballas et al. (2009) reported that MeCP2 deletion was also observed in astrocytes and that MeCP2-deleted astrocytes could not support the normal neuronal growth. By coculturing neuronal cells with FXS astrocytes, Jacobs et al. (2010) found that FXS astrocytes can delay dendritic and synaptic growth. Similarly, Cheng et al. (2016) also demonstrated that neurons showed morphological defects and changes in the synaptic formation of the dendritic spines when neuronal cells were cocultured with FXS astrocytes. In the *Fmr1* knockout mouse model simulating FXS, dendritic developmental changes can also be observed (Hodges et al., 2017). Similarly, DS astrocytes are also toxic to neurons *in vivo* and *in vitro* (Chen et al., 2014). These experiments showed that astrocytes are closely associated with the development and formation of neurons and the occurrence of ID-related diseases, although the roles of astrocytes in ID should be further investigated.

### Reactive Astrocytes in Intellectual Disability

When the CNS is damaged or pathological changes occur, astrocytes will respond to abnormal nerve cells through release of various molecules inside and outside the cells, resulting in haemostatic, morphological, and functional changes and transforming into reactive astrocytes (Liddelow and Barres, 2017). Increased reactive astrocytes will have beneficial or harmful effects on the peripheral nerve and non-nerve cells through functional changes, and reactive astrocytes will change with the degree of nervous system injuries (Boghdadi et al., 2020). Increased reactive astrocytes are common in all kinds of CNS injuries, and increased reactive astrocytes are usually regarded as a pathological marker of CNS injuries. Furthermore, activated astrocyte markers such as GFAP and S100β were increased pathologically (Abdelhak et al., 2022). GFAP and S100β were also increased in astrocytes in various neurological disease models with intellectual impairment symptoms, such as RS, DS (Chen et al., 2014), FXS (Yuskaitis et al., 2010), and TS (Sosunov et al., 2008), indicating that reactive astrocytes are significantly increased in ID-related diseases.

Increased reactive astrocytes in ID-related diseases generally have adverse effects on the CNS, which is generally manifested in the inflammatory response (Sofroniew, 2020). The inflammatory factor expression levels, such as IL-1β, IL-6, and TNF-α, were upregulated in RS astrocytes (Maezawa et al., 2009), the expression of tumour necrosis factor receptor 2 (TNFR2) was significantly increased in FXS astrocytes (Pacey et al., 2015) and the increased expression of immune response regulators miR-146 and miR-155 associated with the inflammatory response in reactive astrocytes was also observed in a DS mouse model (Arena et al., 2017). The inflammatory reaction brought by these reactive astrocytes affects the CNS homeostasis, inhibiting the normal growth or apoptosis of neurons.

### Ion Channel Dysfunction of Astrocytes in Intellectual Disability

In addition to the inflammatory response, astrocytes have many effects on neuronal ion homeostasis in various ID-related diseases. Ion homeostasis is very important for the electrophysiological activities of the neurons. Ion homeostasis imbalance is shown in various neurological diseases and results in abnormal excitation and even neuronal death (Ramocki and Zoghbi, 2008). DS astrocytes frequently show spontaneous calcium fluctuations, resulting in decreased excitability of cocultured neurons (Mizuno et al., 2018); similarly, zinc ion concentration decreases in DS astrocytes, affecting the synaptic transmission function (Ballestín et al., 2014). In the TS model, the expression of K^+^ channels on the astrocyte surface is reduced, which will decrease their ability to buffer potassium, and the gap connection between astrocytes responsible for potassium redistribution would be damaged (Wong, 2019). A series of changes will eventually result in the overexcitation of neurons, leading to TS complex (Jansen et al., 2005). In addition to astrocyte K^+^ channels, the astrocyte ion channels TREK-1 and Bestrophin-1 (BEST-1) mediated the fast and slow glutamate releases in astrocytes and then affected neighbouring neurons (Woo et al., 2012). The connexin hemichannels controlled the ATP and glutamate release and then affected the CNS activity (Xing et al., 2019). Therefore, astrocyte ion channels, such as the K^+^ channel, BEST-1, and hemichannels, contributed to the neurodevelopmental disability and ID (Olsen et al., 2015), although their molecular mechanisms are needed for further investigation. In addition to ion channels, the metabotropic glutamate receptor 3 protein levels associated with calcium metabolism were also down-regulated in RS astrocytes (Pacheco et al., 2017). The ion homeostasis imbalance leads to neuronal damage, and the glutamate metabolism imbalance caused by astrocyte activation is also common in various ID-related diseases. Higashimori et al. (2013) found that the metabotropic glutamate receptor 5 expressions and (s)-3,5-dihydroxyphenylglycine-dependent Ca^2+^ response were decreased in FXS astrocytes, weakening the expression of neuron-dependent GLT1 and eventually reducing the neuronal glutamate uptake. An imbalance between glutamate and GABA in FXS astrocytes produces neurotoxicity, which shortens the neuronal dendrite lengths (Wang et al., 2016). The expression of glutamate transporters in astrocytes, such as GLT-1 and GLAST, also decreased in the TS model (Wong et al., 2003), and astrocytes have increased extracellular glutamate levels, resulting in central nerve cell death and impaired synaptic plasticity and learning due to excitotoxicity (Zeng et al., 2007). However, reactive astrocytes in these diseases are not all unfavourable. Some studies on disease models have found that reactive astrocytes can also protect neurons against CNS damage. In the RS model, Okabe et al. (2012) and Iyer et al. (2014) demonstrated that the expression of metabotropic glutamate receptor 5 increased in RS and DS astrocytes, and the astrocytes enhanced the scavenge of the glutamate, which could prevent neuronal overexcitation caused by glutamate and protect the neurons.

### Molecular Dysfunction of Astrocytes in Intellectual Disability

Changes in molecular pathways of reactive astrocytes are also very common. The expression levels of many proteins in astrocytes of various diseases will also affect their occurrences. In the RS model, decreased brain-derived neurotrophic factor in astrocytes may be one of the reasons leading to abnormal neural development (Maezawa et al., 2009). In the DS model, the sustained high S100β expression in astrocytes will increase the production of reactive oxygen species and activate the stress response kinase, resulting in cell apoptosis (Esposito et al., 2007). The number of calcium-binding presynaptic proteins, synaptophysins, and postsynaptic scaffold proteins, PSD95, located at the neuronal synapses is reduced due to coculture with FXS astrocytes, showing an abnormal dendritic morphology (Jacobs and Doering, 2010). Thrombospondin-1 (TSP-1) is an important astrocyte secretory protein (Ren et al., 2019) that helps regulate spinal development (Torres et al., 2018) and synaptic formation (Christopherson et al., 2005). The TSP-1 protein expression significantly decreased in FXS astrocytes (Cheng et al., 2016). Similarly, decreased TSP-1 protein expression in DS astrocytes may cause an abnormal synaptic formation (Garcia et al., 2010).

Regarding the gene expression of astrocytes, the gene *Slc9a3r1* encoding scaffold protein NHERF1 was upregulated in RS astrocytes, damaging the RNA metabolism, protein homeostasis, monoamine metabolism, and cholesterol synthesis (Pacheco et al., 2017). Furthermore, two genes, *Chgb* and *Lcn2*, encoding the secretory protein chromogranin B and lipocalin-2, were also detected and significantly up- and down-regulated, respectively, in the RS model, and proteins secreted by the RS glial cells may have adverse effects on the dendritic structure of the neurons (Delépine et al., 2015). In DS astrocytes, Chen et al. found that *NFE2L2*, *TSP-1*, and *TSP-2* expression levels were decreased, which also increased the reactive oxygen species levels and decreased the synapse formation in astrocytes (Chen et al., 2014). Furthermore, astrocytes were also involved in the overactivation of the Akt/mTOR axis in DS neurons, which eventually result in neuronal abnormalities and autonomic dysfunctions (Araujo et al., 2018).

### Astrocyte Dysfunction in Environmental Factor-Associated Intellectual Disability

Excessive alcohol intake from environmental factors will also affect the process of intellectual development. Ethanol has been reported to decrease the total protein in astrocytes and suppress the serotonin uptake by astrocytes (Lokhorst and Druse, 1993). This discovery reveals the relationship between ethanol and CNS development. A pregnancy-related infection will also affect the development of astrocytes and neurons. The infection profoundly altered astrocytic biology and increased reactive astrocytes. The offspring of rats infected during pregnancy have higher serotonin receptor sensitivity, which increases the risk of neurological diseases (Wischhof et al., 2015). GFAP and IL-6 expressions were increased in the astrocytes of rat offspring infected during pregnancy, resulting in abnormal myelin development (Huleihel et al., 2004). Recent studies suggested that infection during pregnancy affected neural development, resulting in intellectual impairment and eventually neurodegenerative diseases (Samuelsson et al., 2006), which may be the main molecular mechanisms of intellectual impairment due to pregnancy-related infection (Li et al., 2015). *In vitro* lead exposure experiments show that Pb can activate astrocytes, promote the release of inflammatory factors in astrocytes and cause an inflammatory response (Baruch et al., 2015). Pb can also produce oxidative stress in astrocytes (Qian and Tiffany-Castiglioni, 2003), block the cell cycle of astrocytes (Rahman et al., 2019), induce astrocyte apoptosis, and eventually result in neurodegenerative diseases. Moreover, experiments have proven that Pb can reduce serotonin in the mouse brain and contribute to ID (Park et al., 2016).

## Effects of ID Drugs on Astrocytes

Five approaches have been used for ID management: physical, training, psycho-, dietary, and drug therapy. This mini-review focuses on drug treatment. Multiple drugs have already been used for the treatment of mental disorders, such as piracetam, aniracetam, monosialotetrahexosylganglioside sodium (GMI), mecobalamin, vitamin B12, and methylphenidate hydrochloride.

Cetam drugs can reverse the effects of scopolamine of reducing glucose utilisation in the cerebral cortex (Piercey et al., 1987). In the Alzheimer’s disease model, mitochondrial ATP production and membrane potential were enhanced after the piracetam treatment, which reduced apoptosis and finally enhanced neuronal plasticity (Leuner et al., 2010). Simultaneously, studies have shown that cetam drugs can slow down the inactivation of the AMPA receptor (Jin et al., 2005) and increase the calcium level in astrocytes (Marisco et al., 2013). Slowing the AMPA receptor inactivation can also block the potassium current in astrocytes by increasing an endogenous Na^+^ level (Robert and Magistretti, 1997) and protect against the toxicity caused by neuronal overexcitation. Cetam drugs can also reduce the oxidative stress damage in astrocytes (Gabryel et al., 2002) and reduce the GFAP and reactivity of astrocytes (Pimentel et al., 2009) to improve patients’ memory and brain function (Malykh and Sadaie, 2010).

Monosialotetrahexosylganglioside sodium (GMI) is an important component of the mammalian nerve cells (Wang et al., 2021) and can partially prevent an abnormal release or reuptake of excitatory amino acid neurotransmitters, such as glutamate (Zhang et al., 2015); thus, it can inhibit excessive glutamate expression from damaging brain nerves and tissues (Hinzman et al., 2012). GMI can also improve cerebral blood circulation, increase blood oxygen, protect neurons (Aureli et al., 2016) and reduce *N*-methyl-D-aspartate-dependent excitotoxicity from astrocyte morphological swelling (Häussinger and Schliess, 2005).

Vitamin B12 was needed for nerve development (Selhub et al., 2000). Reactive astrocytes were increased in the grey matter of vitamin-B-deficient rats, accompanied by microglial oedema and myelinolytic lesions (Scalabrino, 2009). Mecobalamin and vitamin B12 can supplement vitamin B12 in the brain; promote nucleic acid and protein synthesis; enhance axonal transportation, axonal regeneration, and myelin formation to prevent axonal degeneration; repair damaged nerve tissues; and improve the damaged glial cells in the brain (Wu et al., 2019).

Furthermore, a previous study reported that the antibiotic minocycline can partially correct the pathological phenotypes of astrocytes by specifically regulating the S100β, GFAP, inducible nitric oxide synthase and thrombospondins 1 and 2 expressions in DS astrocytes (Chen et al., 2014), which may provide a new treatment for DS.

## Summary and Prospect

With an in-depth investigation of astrocytes in recent years, their roles in neural development have been gradually explored. Their changes are also closely associated with the occurrence and progression of many neurological diseases. The aetiology of ID is complex, and several mechanisms remain unclear. Therefore, this mini-review shows that functional changes of astrocytes under the action of various pathogenic factors are closely related to ID, and various drugs for ID can also attenuate astrocyte lesions to varying degrees. However, a few effective drugs have been administered for functional changes of astrocytes under pathological conditions to treat ID-related diseases. This suggests that morphological and functional changes of astrocytes may be one of the important mechanisms of these diseases. Dissecting the contribution of distinct astrocyte subtypes to ID-related diseases might provide a potential therapeutic approach and benefit these patients. Therefore, drugs that inhibit astrocyte lesions may become a new and important treatment strategy for ID in the future.

## Author Contributions

BW and LZh wrote the manuscript. BW, LZo, ML, and LZh reviewed the manuscript. All authors contributed to the article and approved the submitted version.

## Conflict of Interest

The authors declare that the research was conducted in the absence of any commercial or financial relationships that could be construed as a potential conflict of interest.

## Publisher’s Note

All claims expressed in this article are solely those of the authors and do not necessarily represent those of their affiliated organizations, or those of the publisher, the editors and the reviewers. Any product that may be evaluated in this article, or claim that may be made by its manufacturer, is not guaranteed or endorsed by the publisher.
