# Supplementary figures and images for: Astrocyte: A Foe or a Friend in Intellectual Disability-Related Diseases
Source: Front Synaptic Neurosci. 2022 Jun 23;14:877928. doi: 10.3389/fnsyn.2022.877928 (PMC9259964; doi:10.3389/fnsyn.2022.877928)

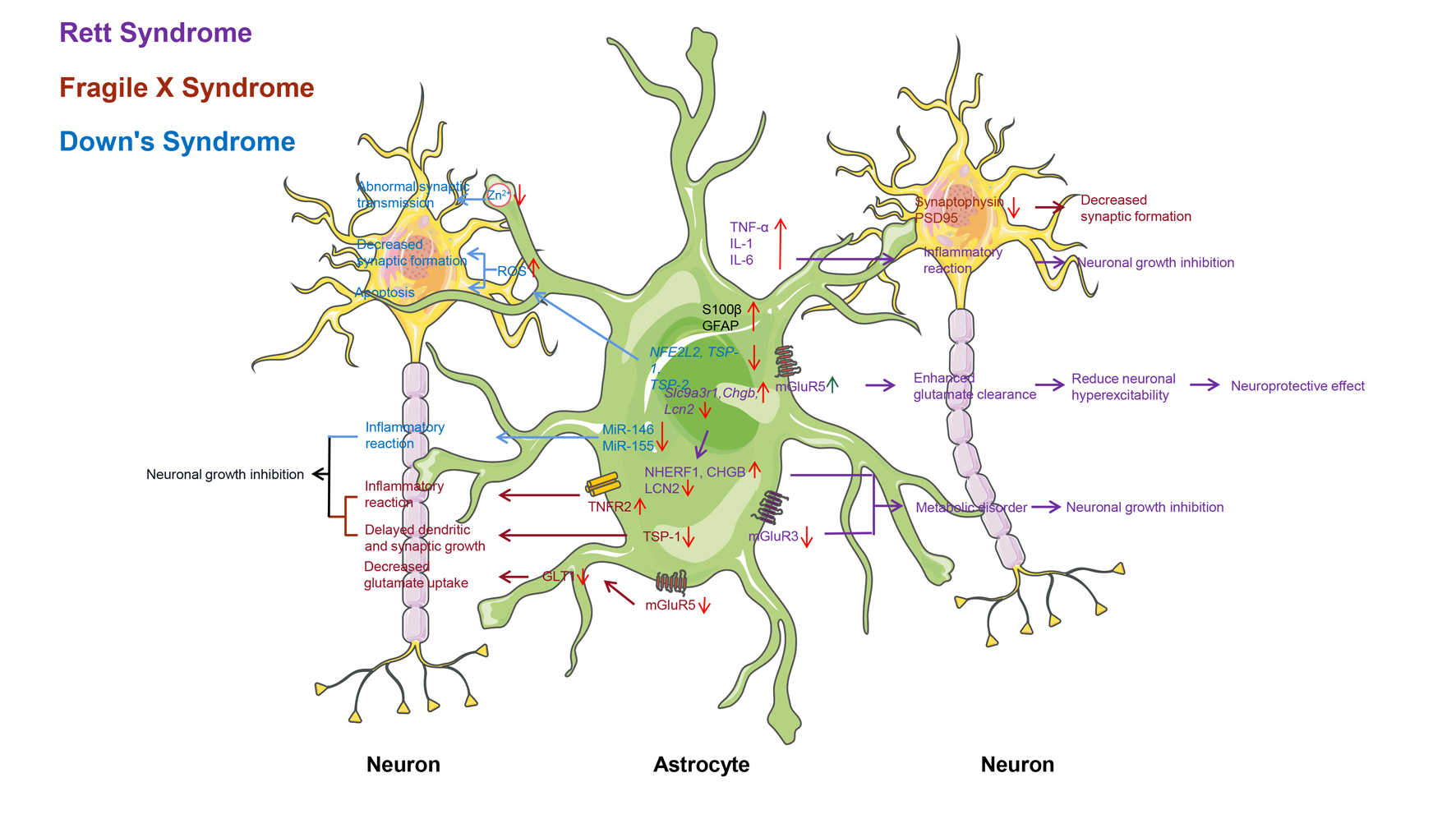

Supplement: Supplementary file 1 [file Image_1.TIF]
